# The Effectiveness of Non-Soy Oral Herbal Supplements for Menopausal Symptoms: A Systematic Review

**DOI:** 10.3390/nu18132037

**Published:** 2026-06-23

**Authors:** Grace Jing, Clara M. Keane, Clare M. Reynolds

**Affiliations:** 1 School of Agriculture and Food Science, University College Dublin, D04 V1W8 Dublin, Ireland; grace.jing@ucdconnect.ie (G.J.); clara.keane1@ucdconnect.ie (C.M.K.); 2 School of Public Health, Physiotherapy and Sports Science, Discipline of Dietetics/Conway Institute, University College Dublin, D04 V1W8 Dublin, Ireland

**Keywords:** phytoestrogen, menopause, herbal treatment, menopausal symptoms, menopausal quality of life

## Abstract

**Background**: Menopause is a biological process characterised by the cessation of menstruation and substantial changes in ovarian hormone production. As many women seek non-hormonal alternatives to hormone replacement therapy, interest in herbal supplements has increased. However, the evidence for their effectiveness remains inconsistent due to the diversity of available supplements. This systematic review aimed to evaluate whether oral herbal supplements improve menopausal symptoms in women. **Methods**: A systematic search of PubMed, Scopus, Embase and PsycINFO was conducted in October 2025 to identify randomised controlled trials (RCTs) assessing non-soy oral herbal supplements for menopausal symptoms. The primary outcomes examined included neurological symptoms, sleep quality, and vasomotor symptoms. The secondary outcomes were overall quality of life and reported adverse effects. **Results**: Thirty RCTs met the inclusion criteria. Of these, 28 reported improvements in menopausal symptoms, particularly reductions in the severity and frequency of vasomotor symptoms. Nineteen distinct herbal interventions were identified. The two RCTs using black cohosh and dong quai found no measurable effects. Two studies demonstrated significant improvements in specific cognitive domains, although evidence for broader cognitive benefits was limited. Overall quality of life was generally improved, and no major adverse effects were reported. **Conclusions**: The substantial heterogeneity in the studies identified from this review across multiple factors, including different herbal interventions, doses, duration, and assessment methods, makes it difficult to come to an overall conclusion on whether or not herbal supplementation is effective for treating menopausal symptoms. Further well-designed trials are needed to clarify the efficacy and optimal dosing of herbal treatments.

## 1. Introduction

Menopause is a natural biological transition marked by the cessation of menstruation and is accompanied by physiological changes and symptoms arising from the reduction in hormones in the body. According to the Stages of Reproductive Ageing Workshop (STRAW) [1], this is a staged process which includes a transition from perimenopause, which is characterised by irregular menstrual cycles and rising follicle-stimulating hormone (FSH) concentrations, to menopause, characterised by the cessation of menstruation; and then to post menopause, characterised by reduced oestrogen and progesterone and high concentrations of FSH [2].

Oestrogen is a critical hormone in various biological processes, including protein synthesis, regulation of reproductive health, and supporting the brain, heart, and musculoskeletal health [3]. The decline in oestrogen results in a wide range of symptoms that can have a dramatic impact on quality of life [4]. Generally, there are four interlinked categories of menopausal symptoms: vasomotor symptoms, vaginal dryness, sleep difficulties and psychological impacts [5]. While the causative mechanisms that underlie these effects can be attributed to declining oestrogen concentrations, the exact molecular mechanisms have not been fully elucidated.

The cumulative health burden associated with these symptoms is substantial, particularly as global life expectancy increases and more women spend longer years in the postmenopausal stage [6]. Hormone replacement therapy (HRT) has long been considered the most effective treatment for menopausal symptoms; however, there is some evidence demonstrating risks in certain individuals, particularly in relation to stroke and thrombosis [7]. The British Menopause Society has recommended low-dose natural phytoestrogens as a safer alternative treatment for menopausal symptoms [8]. Despite interpersonal differences, some studies show that herbal supplementation has positive effects like reducing vasomotor symptoms, sleep disturbances, vaginal symptoms, and improving mood, well-being and the overall quality of life in menopausal women [9].

Many of these herbs contain phytoestrogenic compounds. These bioactives differ structurally from oestrogen but have the ability to bind to β-oestrogen receptors [10]. Phytoestrogens are increasingly recognised for their role in maintaining hormonal balance and benefiting menopausal women through mood regulation, neurological regulation, cardiovascular protection, bone strengthening and thermoregulation [11].

A number of studies have examined the effectiveness of medicinal herbs in treating menopausal symptoms, their mechanism of action and any adverse effects that may be associated with them, but the results are often contradictory. This is possibly due to the different methods of herbal preparations, variability in active constituents, and other confounding factors such as treatment duration, which complicates the process of establishing clear therapeutic guidelines. Additionally, numerous herbal supplement options exist on the market, which may be overwhelming for individuals seeking menopause support.

The aim of this review was to evaluate whether oral herbal supplements have positive effects on the menopausal symptoms of women, such as vasomotor symptoms (such as hot flashes and night sweats), neurological symptoms (such as mood and cognition), sleep quality, quality of life measures and adverse effects.

## 2. Materials and Methods

### 2.1. Protocol

A protocol for this systematic review was developed a priori in accordance with the Preferred Reporting Items for Systematic Reviews and Meta-Analyses (PRISMA) guidelines and guided all stages of the review process; however, it was not registered in PROSPERO [12].

### 2.2. Eligibility Criteria

PICOS (population, intervention, comparator, outcomes, and study design) criteria were utilised when establishing the exclusion and inclusion criteria (see Table 1). Soy-based interventions were excluded because soy isoflavones represent a distinct and extensively studied category of dietary phytoestrogen therapies in menopause, often addressed in dedicated systematic reviews [13,14]. Their inclusion would have substantially broadened the scope of this review and diverted focus from non-soy herbal supplements.

### 2.3. Search Methods

Searches were conducted between 1 October 2025 and 11 October 2025. Information sources used included four online databases: PubMed, PsycINFO, EMBASE and Scopus. A search protocol with keywords and medical subject headings (MeSH Terms) was developed to identify relevant studies, and the search string was slightly adapted for each database (Supplementary Materials). All relevant studies identified were then imported into EndNote as references and then imported into Covidence for abstract and full-text screening. The screening process was done independently by two reviewers, C.M.R. and G.J. Where a conflict arose, a meeting was held to discuss the final decision.

### 2.4. Data Extraction

Data extraction was performed by one reviewer (G.J.), independently using Microsoft Excel (Table in Section 3.2). A total of 20% of papers were checked by C.M.R. to ensure consistency and reproducibility. Relevant items included first author and year of publication, study design, duration, sample size, intervention and control group, setting, outcomes and main findings. The results were reported narratively.

### 2.5. Risk-of-Bias Assessment

All eligible RCT studies were then assessed for risk of bias using the Cochrane Risk of Bias Tool 2.0 (RoB2) under the five domains [15] and were classified as either low risk of bias, having some concerns or high risk of bias. Studies were not excluded based on their risk of bias.

## 3. Results

### 3.1. Study Selection

**Figure 1:**
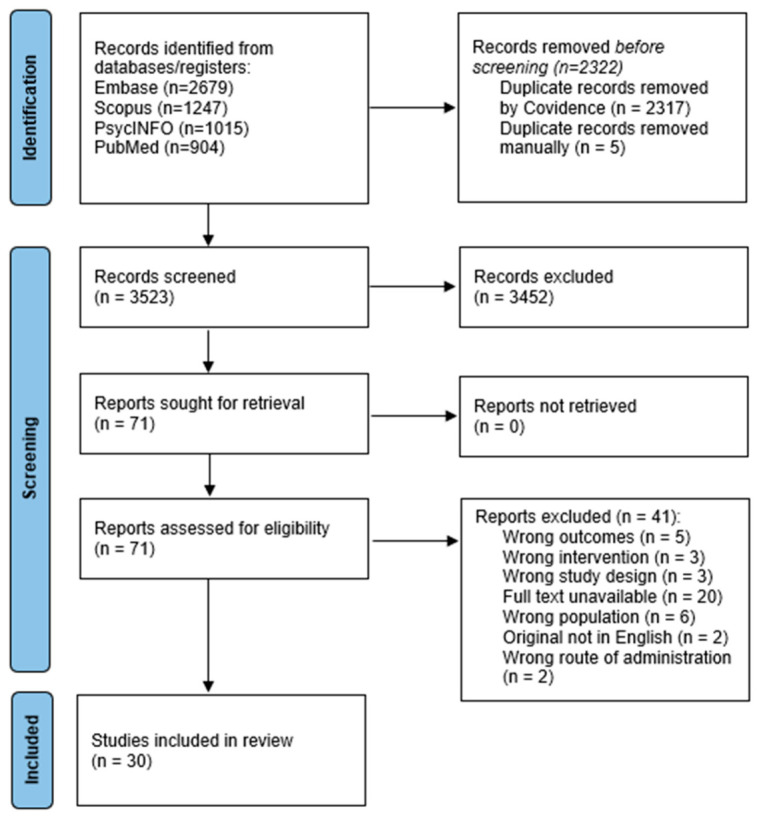
PRISMA flow diagram illustrating the abstract screening process and reasons for full-text exclusion.

The systematic literature search captured 5845 studies across the four databases. After full-text screening, a total of 30 studies published between 1997 and 2024 were considered as eligible for this systematic review, involving 2541 participants. The details of the duplicated papers and reasons for exclusion are shown in Figure 1.

### 3.2. Data Extraction of Studies

Table 2 is a summary of all the relevant studies. A total of nineteen different herbal supplements were identified, of which the most common were black cohosh (*Cimicifuga racemosa*; four studies) [16,17,18,19], evening primrose oil (four studies) [20,21,22,23], hop (*Humulus lupulus*; three studies) [24,25,26], red clover (three studies) [27,28,29], sage (*Salvia officinalis*; two studies) [30,31] and valerian (two studies) [32,33]. Other herbal interventions included yam (*Diascorea alata*) [34], fennel [21], jujube seed [35], liquorice [36], basil (*Ocimum basilicum*) [37], pomegranate [38], French maritime pine extract, rhubarb (*Rheum ribes* root) [39], five-flavour berry (*Schisandra chinensis*) [40], milk thistle (*Silybum marianum*) [41], *Sophora japonica* fruit extract [42], St John’s wort (*Hypericum perforatum*) [43], and dong quai [44]. The population was primarily healthy peri-menopausal, menopausal, and post-menopausal women aged over 40 years. The study duration widely ranged from 3 weeks to 12 months. The most common tools used to measure the outcomes were Kupperman’s Index (KI), the Menopause-Specific Quality of Life Questionnaire (MENQOL), the Greene Climacteric Scale (GCS), the Menopause Rating Scale (MRS), the Hot Flush Severity Score (HFS), and the Pittsburgh Sleep Quality Index (PSQI).

### 3.3. Menopause-Related Subjective Questionnaires

#### 3.3.1. Kupperman’s Index (KI)

KI is a 13-item questionnaire assessing menopausal symptoms, including sweating, hot flashes, insomnia, melancholia, nervousness, palpitation, sexual complaints and urinary tract symptoms [47], and was used in 13 studies [18,25,26,27,29,32,38,39,40,42,43]. The herbal supplements used were pomegranate, St John’s wort, hop, dong quai, valerian, sophora japonica fruit extract, red clover, five-flavour berry (*Schisandra chinensis*), black cohosh and rhubarb. Eleven of these studies reported significant improvements in the overall KI scores in the intervention groups compared with placebo. These improvements were particularly evident in the vasomotor and psychological symptom domains. However, two studies reported no significant changes; these studies investigated dong quai and black cohosh [19,44].

#### 3.3.2. Greene Climacteric Scale (GCS)

Four studies used GCS, which is a scale composed of 21 questions, measuring menopausal-related mental (anxiety and depression), physical and vasomotor symptoms, all of which reported significant benefits after supplementation in total GCS scores and with analysis of subcategories from the GCS [17,24,34,41]. The herbal supplements used in the four studies were hop, yam (*Diascorea alata*), milk thistle (*Silybum marianum*) and black cohosh.

#### 3.3.3. Menopause Rating Scale (MRS)

The MRS is an 11-item tool covering three menopausal symptom categories—somatic, psychological and urogenital symptoms—and was used in five studies, all of which reported significant differences between the intervention and placebo groups (*p* < 0.05) using basil, fennel, evening primrose oil (EPO) and Salvia officinalis [21,23,30,31,37]. An additional study used MRS in combination with KI and did not see a significant difference in the MRS scores between the placebo and hop supplement groups (*p* = 0.06) [26].

#### 3.3.4. Other Measurement Questionnaires

Other minority tools were used to assess hot flashes. This included the Hot Flush Severity Score (HFS), which was used by Wilfried et al. [30] and showed a 55.3% reduction in the score (*p* < 0.028). A common method to assess vasomotor symptoms was a hot flush self-reported diary; this was used in three studies examining hop [25], dong quoi [44] and red clover [29] and demonstrated significant benefits in the supplement groups compared with placebo. Hot flashes were also assessed using the Wiklund Vasomotor Symptom Subscale score in a study that examined EPO supplementation and found that while there was no difference in hot flashes, the incidence of night sweats was reduced [22]. Women’s Health Questionnaire (WHQ) was used to record symptoms following supplementation with Pycnogenol (French Maritime Bark extract) and showed significant reduction across climacteric symptoms [46]. The Hot Flash-Related Daily Interference Scale (HFRDIS) was used to assess the impact of EPO supplementation on QoL and showed significant differences in several subdomains including social activities, relations with others and sexuality [20]. Mirabi et al. collected information on hot flash incidence and severity in response to valerian root extract supplementation using a researcher-developed questionnaire [33] and found a significant reduction in the incidence and severity of hot flashes. Similarly, Nahidi used a researcher-developed questionnaire and found significant differences in hot flash incidence during the supplementation period and for 2 weeks following follow-up [36].

#### 3.3.5. Sleep Specific Outcomes

Four studies reported specific sleep-related outcomes using tools such as the Pittsburgh Sleep Quality Index (PSQI) and quantitative electroencephalography (qEEG). Jiang et al. found that black cohosh supplementation led to non-significant polysomnographic changes (*p* = 0.051) after 6 months [45]; Zeidabadi et al. and Mahmoudi et al. reported that the mean score of PSQI significantly improved in the intervention groups taking sage or jujube seed supplements, respectively (*p* < 0.05 and *p* < 0.001, respectively) [31,35], while Wilfried et al. found a positive impact of taking sage extract for 4 weeks on sleep quality, using qEEG (*p* < 0.05) [30].

#### 3.3.6. Neurological Outcomes

One study used the Hamilton Depression Rating Scale (HDRS) to assess the mood of participants and found that 80% of women in the intervention group taking St John’s wort (otherwise known as Hypericum perforatum) did not display depressive symptoms (*p* < 0.001) [43]. Moreover, the only study using cognitive-specific questionnaires concluded that red clover supplementation improved the results of block design tests in menopausal women, but no other major short-term cognitive effects were seen [28]. Additionally, Yang et al. demonstrated that taking French maritime pine bark extract for 6 months resulted in improved self-reported memory, reported as a subsection of the WHQ [46].

#### 3.3.7. Hormonal Analysis

Two studies measured hormonal profiles. Hsu et al. concluded that compared with the placebo group, there were positive effects on serum follicle-stimulating hormone (FSH) (*p* = 0.009) and oestradiol (*p* = 0.043) concentrations after 12 months of Diascorea treatment [34]. Similarly, Ghavi et al. also found significant positive changes in the mean FSH and oestradiol levels in both of their intervention groups after 8 weeks of fennel or EPO supplementation (*p* < 0.001) [21].

### 3.4. Quality of Life (QoL)

The Menopause-Specific Quality of Life Questionnaire includes four subsets of questionnaires: vasomotor, psychosocial status, physical status and sexual status. Out of the four studies that used the MENQOL, one found no improvement after taking black cohosh for 12 weeks [17]; one found improvements in the vasomotor and physical domains only after 6 months of black cohosh supplementation; and two show significant improvements (*p* < 0.05) after either 4 weeks of pomegranate [38] or 12 weeks of black cohosh treatment [18].

Most papers did not report adverse effects or only reported minor adverse effects, which were relieved after 1–3 days, which was considered an unlikely result of the intervention. However, Schellenberg et al. reported that 21 nonserious adverse events occurred in 20 patients, nine of which were possibly treatment-related, after 12 weeks of black cohosh supplementation [18].

**Figure 2:**
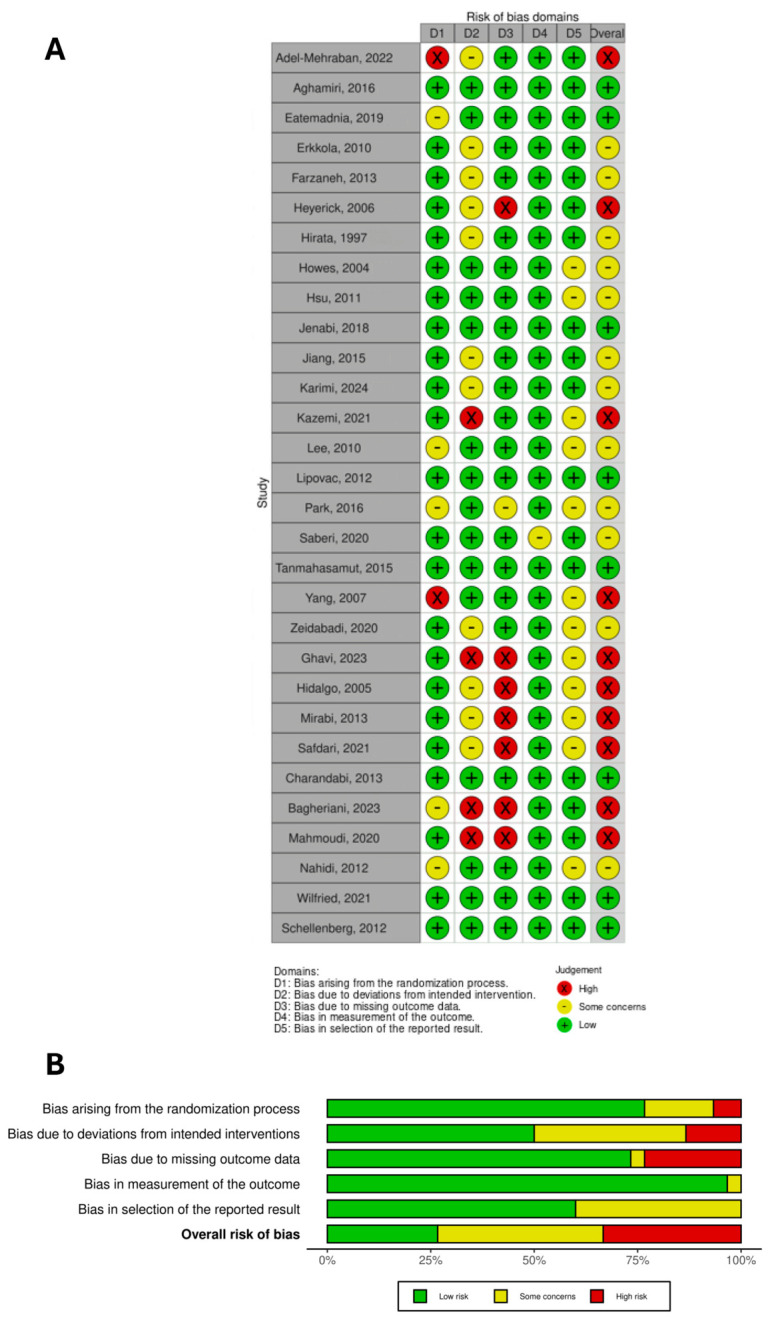
The overall risk of bias and the risk of bias for each domain of the eligible papers (**A**), and the details of risk of bias for each eligible study (**B**), assessed using the Cochrane Risk of Bias Tool 2.0 (RoB2) [17,18,19,20,21,22,23,24,25,26,27,28,29,30,31,32,33,34,35,36,37,38,39,40,41,42,43,44,45,46].

Out of the 30 studies, 10 studies were characterised as having a high risk of bias; 12 studies had some concerns; and eight studies were classified as having a low risk of bias (see Figure 2A,B). Across the five risk-of-bias domains, most studies were judged as having some concerns, particularly for deviation from the intended study and selective reporting. Bias in measurement of the outcome remained the lowest. A high risk of bias was mostly associated with the randomisation process, deviation from the intended interventions and missing outcome data.

## 4. Discussion

Herbal interventions for menopausal symptoms sit within the broader research field of phytotherapy, an area grounded in traditional herbal medicine that has historically been used to manage conditions ranging from acute illnesses to chronic diseases for both men and women. The bioactive phytocompounds present in plants, including steroids, tannins, and flavonoids, are extensively used by practitioners and may have antimicrobial, anti-inflammatory, antioxidant, neuroprotective, cardioprotective and immunomodulatory properties [48]. Given the long history of herbal medicine, many herbs with or without phytoestrogenic properties have been studied for their potential to alleviate symptoms experienced by menopausal women.

Evidence shows that phytoestrogens have other benefits, including the prevention of osteoporosis and protecting women from oestrogen carcinogenesis, as they are aromatase inhibitors and possess antioxidant and anti-inflammatory properties [49]. The studies presented in this review showed a considerable range of supplements, and the variation in their effectiveness may be due to different bioactive mechanisms. However, the strength of mechanistic evidence varied between herbal supplements, and some proposed pathways remain uncertain due to limited clinical validation. For example, black cohosh has both oestrogenic and serotonergic effects [49]. Black cohosh is also known to have vasorelaxant qualities that may confer therapeutic value [16]. Indeed, three of the four black cohosh studies in this review reported beneficial effects on menopausal symptoms, including increased sleep quality, vasomotor and physical symptoms. An additional study utilising an extract of black cohosh (Ze 450) was also identified; this study demonstrated a dose-dependent reduction in the severity of symptoms and QoL using the KI tool [18]. However, the risk of bias was low in only three of these studies, and the evidence should be interpreted with caution. Furthermore, given the heterogeneity in dose (studies ranged between 2.5 g and 40 mg) and questionnaires used to assess symptoms (KI, GCS, MENQoL, and PSQI), it is not possible to make any recommendation in relation to the use of black cohosh as a supplement for treatment of menopausal symptoms. This aligns with the broader literature. A previous systematic review concluded that there is insufficient evidence to support the use of black cohosh for menopausal symptoms, and recent menopause guidance does not recommend black cohosh for vasomotor symptoms because of inconsistent efficacy data [50]. An additional limitation is that black cohosh preparations are not always chemically equivalent; extracts may differ in plant part, extraction method, standardisation of active constituents, and commercial formulation. Therefore, findings from one preparation, such as Ze 450, may not be generalisable to all black cohosh products. Although black cohosh was generally well tolerated in the trials included in this review, rare cases of liver injury have been reported in association with products labelled as black cohosh [51], meaning safety conclusions should also be interpreted cautiously.

Other oestrogenic supplements were identified in this review. Red clover contains bioactive isoflavones such as biochanin A, formononetin, daidzein and genistein. These compounds possess structural similarities to oestradiol and can weakly bind and activate oestrogen receptors [52]. Three studies examined red clover supplementation for menopausal symptoms. Howes et al. specifically investigated cognitive outcomes using aglycone isoflavones derived from red clover [28]. Although a small improvement was observed in block design test performance, worsening was reported in digit recall and verbal memory assessments, leading the authors to conclude that there were no overall cognitive benefits after 6 months of supplementation. In contrast, two studies examining 80 mg of red clover supplementation reported significant reductions in vasomotor symptom frequency and severity using the KI questionnaire [27,29]. Collectively, these findings suggest that any benefits associated with red clover may be symptom-specific, with more consistent evidence for vasomotor symptoms than cognitive outcomes. However, evidence remains limited by the small number of studies, variation in formulations, and uncertainty regarding the clinical significance of phytoestrogen-mediated mechanisms.

In addition to red clover, this review also identified studies examining hops. These studies were specifically focused on extracts that were enriched for 8-prenylnarigenin. This is a prenylated flavonoid phytoestrogen which can bind to both the alpha and beta oestrogen receptor. While it binds more weakly than oestradiol, it is thought to compensate when oestradiol concentrations begin to decline. Of the studies that examined climacteric effects, hop supplementation appeared to be more consistent, with both studies demonstrating significant improvements in vasomotor menopausal symptoms as well as beneficial effects in relation to sleep quality and fatigue [24,26]. This is supported by other studies, albeit mechanistic pre-clinical studies, that demonstrated that the activation of GABA receptors by bioactives such as humulone in hops can promote sleep [53,54]. The mechanistic understanding in relation to menopausal symptoms was not investigated, but it may be possible that it is a combination of multiple molecular pathways that contribute to the observed effects. However, as with other supplements, factors such as small sample sizes, variation in outcome measures and, in some cases, high risk of bias make it difficult to generalise these findings.

In addition to these more commonly studied supplements, this review identified several other individual studies examining other oestrogenic compounds. This included fennel, which contains oestrogenic bioactives, including anethole, dianethole and photoanethole, and demonstrated alterations in FSH and oestradiol as well as a reduction in the MRS score [21]. Pomegranate was also used as a supplement; however, the bioactive compounds such as ellagitannins, polyphenols and lignans are weaker oestrogen receptor agonists than those found in hops or red clover. Pomegranate is likely to activate multiple molecular pathways (inflammatory, antioxidant, etc.) rather than reliance on oestrogenic effects. A recent scoping review showed reduced testosterone, oxidative stress and inflammation in women with PCOS as well as positive effects on bone formation and vaginal atrophy in a murine ovariectomy model [55]. Interestingly, supplementation resulted in reduced menopausal symptoms and increased QoL [38]. Similarly, liquorice and rhubarb may exert beneficial effects through multiple pathways beyond simple phytoestrogenic activity. Liquorice contains flavonoids capable of weak oestrogen receptor modulation alongside potential effects on cortisol metabolism and inflammatory signalling [36], whereas rhubarb contains stilbene compounds with proposed selective oestrogen receptor modulatory activity that may influence thermoregulation and vasomotor symptoms. Indeed, these effects have also been demonstrated in a pre-clinical mouse model [56]. Yams were also used as a supplement to alleviate menopausal symptoms, with one study finding improved GCS scores as well as alterations in FSH and oestradiol concentrations [34]. While yams are known to contain the oestrogenic diosgenin, there is evidence to suggest that this bioactive does not have strong oestrogen receptor effects in reproductive tissues [57]. Similarly, supplementation with milk thistle or sophoro janoica fruit resulted in significant alleviation of menopausal symptoms. While there is some suggestion that these supplements have oestrogenic activity, it is more likely that their effects are due to their anti-inflammatory and antioxidant effects. Collectively, these studies highlight the complexity of interpreting proposed phytoestrogenic mechanisms in menopause. Across these interventions, evidence was typically limited to single studies with relatively small sample sizes, heterogeneity in outcome measures, and, in some cases, high risk of bias, limiting confidence in the generalisability of these findings. Therefore, although these interventions may show promise, current evidence remains insufficient to determine whether they represent effective alternatives for the management of menopausal symptoms.

Additionally, several supplements without oestrogenic effects were identified. EPO contains linoleic acid and γ-linolenic acid, which are converted into prostaglandins (particularly PGE1) and regulate physiological processes such as temperature control, nerve sensitivity and inflammation. EPO is widely used as a supplement in rheumatic diseases, dermatitis and menopausal symptoms [23]. However, it is important to note that while these mechanisms are biologically plausible, the understanding of how these compounds impact menopausal symptoms is limited. Four studies examining EPO were identified in this review; however, there were conflicting results. Of the four studies identified, several different self-reporting tools were used. MRS was used for two of these studies; however, the dose and formulation were not consistent (140 mg of GLA extracted from EPO versus 2000 mg of EPO). While dose relationships should be considered, the studies identified did not show greater impacts with higher doses. There was some evidence of moderate effects on hot flushes and night sweats, as well as alterations in both FSH and oestradiol concentrations and improvement of psychological and sexual aspects of quality of life. The clinical implications of these changes in hormonal concentrations were not defined. When interpreting these studies, the geographical location must also be considered, as all four studies were carried out in Iran, which may limit translatability. Furthermore, three of the studies were considered to be of high risk of bias, with the remaining study noted as having some concerns. It is also worth noting that these studies had relatively small sample sizes, which can further reduce confidence in the generalisability of these results. Taken together, methodological limitations, geographical concentration, heterogeneity in dosing and outcome assessment, and risk of bias substantially limit confidence in the evidence base supporting EPO supplementation for menopausal symptoms [58].

Valerenic acid from valerian root, thujone from sage extracts, and flavonoids from jujube seeds have been proposed to modulate γ-aminobutyric acid (GABA) signalling pathways [48], potentially contributing to anxiolytic, sedative, and neuroprotective effects. Although studies investigating these supplements generally reported improvements in menopausal symptoms, particularly sleep-related outcomes, direct evidence linking these mechanistic pathways to clinical benefit remains limited.

One other key source of heterogeneity, other than the intervention used, was the overlap and inconsistency in the measurement scales used across studies. Although variability in measurement tools makes comparison challenging, multidimensional questionnaires appear to be more informative for capturing the complexity of menopausal symptoms. One narrative review comparing four commonly used questionnaires, KI, MRS, MENQOL and GCS, concluded that although all except KI have undergone validation and have been translated into different languages, notable limitations remain. These include insufficient consideration of ethnic and cultural differences and the absence of clearly defined thresholds for initiating and monitoring treatment [59]. Although they have been compared in terms of structure, validity and applicability, current evidence does not clearly identify a single superior measurement tool. However, MRS and MENQOL demonstrate high methodological quality, with good reliability and validity, and MRS is also suitable for comparisons between cohorts from different countries; GCS, meanwhile, has also been used in recent research to show associations between its symptoms score, body mass index and the risk of developing osteoporosis [59]. Ultimately, the choice of questionnaire depends on the study aims, symptom domains of interest and practical considerations. One aspect that should also be considered is that these scales are self-reported and may, therefore, be subject to reporting bias, recall bias and individual differences in symptom perception and interpretation. Only two of the studies included in this review had biochemical insights, with many studies solely relying on questionnaires.

Across the included studies, comparison between the same herbal supplementation cannot be made due to substantial variability in dosage, extraction methods, and standardisation of the active compound, highlighting the importance of phytochemical consistency in herbal research. Differences in bioavailability and metabolism further complicate interpretation, as many phytoestrogenic compounds require extensive metabolism before becoming biologically active and may, therefore, produce heterogeneous responses between individuals. Furthermore, direct comparisons with HRT were also not possible, as none of the studies included an HRT reference group. Although evidence from an excluded US RCT paper suggests that herbal treatments generally produce milder effects than those typically reported for HRT [60], herbal therapy may still have value for women seeking alternative treatments to HRT. The absence of direct comparative trials remains a major research gap.

More broadly, this review highlights a clear research gap regarding the effects of herbal supplementation on cognitive functions, mood and sleep quality, with several included trials representing the first study of a herb, for example, the Schisandra chinensis study in 2016 [40]. What was clear was the lack of data in relation to neurological symptoms such as mood and cognition and sleep quality, with vasomotor symptoms representing the predominant focus of the identified studies. Future research in this area should incorporate dietary information of the participants to further understand the relationship between dietary supplements and a wider variety of menopausal symptoms. Also, dose-dependent interventions should be used in order to find the optimum dosage for the herbal treatments. Larger sample sizes with more ethnically diverse groups, as well as older women in post-menopausal stages, should be included. Longer study durations with follow-up assessments are also necessary to establish long-term safety and adverse effect profiles. Ideally, studies should also include objective measures, such as blood tests or hormonal assays (e.g., oestrogen levels), and consideration of bioavailability, stability, and biodiversity preservation would strengthen the evidence base for herbal therapies in menopause.

Participant characteristics also limit the generalisability of findings. Most samples consisted of well-educated women with sufficient literacy to complete symptom questionnaires, potentially excluding populations with lower literacy or different socioeconomic backgrounds. Geographically, although this review included studies from multiple regions worldwide, a substantial proportion of the included trials were conducted in Iran, with comparatively fewer studies from Europe, Australia, East Asia, North America, and South America. This uneven geographical distribution introduces the possibility of regional or cultural bias, as menopausal symptom perception, dietary patterns, healthcare practices, and genetic factors may influence both symptom presentation and responsiveness to herbal interventions. Consequently, the findings may not be fully generalisable to the broader global menopausal population. Future studies should aim to include more geographically and ethnically diverse populations to improve external validity.

## 5. Conclusions

Overall, this systematic review identified evidence suggesting that certain herbal supplementations may have the potential to improve some menopausal symptoms, particularly vasomotor symptoms and aspects of sleep and quality of life. However, interpretation of these findings should be cautious given the substantial heterogeneity across interventions, study populations, formulations, outcome measures and methodological approaches. Furthermore, the presence of studies with high or unclear risk of bias, relatively small sample sizes, geographical concentration, and reliance on predominantly self-reported outcomes substantially limits confidence in the evidence base.

Importantly, the findings do not support broad conclusions regarding the clinical effectiveness, comparative efficacy, or long-term safety of herbal supplementation for menopausal symptom management. Although some interventions demonstrated biologically plausible mechanisms, current evidence remains insufficient to recommend specific herbal interventions, formulations or dosing strategies. Future research should prioritise adequately powered randomised controlled trials using standardised preparations, objective outcome measures, longer follow-up periods and more geographically diverse populations. Improving methodological consistency and evidence certainty will be essential before the role of herbal supplementation in menopausal management can be more clearly established.

## Figures and Tables

**Table 1:** Eligibility criteria based on PICOS terms.

PICOS	Inclusion Criteria	Exclusion Criteria
Population	Perimenopausal women aged 40+ experiencing menopausal symptoms. Post-menopausal women aged 40+.	Menopausal women below the age of 40. Women with menopause induced by surgical procedures (e.g., oophorectomy and hysterectomy) or medical conditions (e.g., chemotherapy and autoimmune disorders). Studies conducted in animal models.
Intervention	Oral supplementation.	Non-oral supplements (e.g., cream or ointment). Soy-based interventions.
Comparator	Placebo	Studies that tested on mixed herb formulas.
Outcome	Primary outcomes: Vasomotor symptoms (hot flushes and night sweats). Neurological symptoms (mood and cognition) Sleep quality. Secondary outcomes: Quality of life. Adverse effects.	Sexual dysfunction. Studies that presented results only in terms of quality of life without further explanation.
Study design	Randomised controlled trials (RCTs). Human studies. Peer-reviewed journal articles published in English.	Review papers. Clinical trial registries. Animal studies. Published in a language other than English.

**Table 2:** Study characteristics including first author, year of publication, study design, study duration, sample size, intervention and placebo groups, setting, outcome measured (abbreviation in brackets indicating the appropriate measuring tool used; see footnote for full names), and the main findings of this study.

Author(s)	Study Design	Duration	Sample Size	Intervention/Control Group	Setting	Menopausal Outcomes	Main Findings
Adel-Mehraban, 2022 [38]	RCT	4 weeks	78 healthy volunteer women aged 45–55 yrs	Intervention: 787.5 mg of gallic acid equivalent/g (*n* = 39) Control: placebo (*n* = 39)	Iran	KI and MENQOL	Supplementation reduced KI score and MENQoL (*p* < 0.001). No adverse effects reported.
Aghamiri, 2016 [24]	RCT	12 weeks	120 postmenopausal women and premenopausal women, aged 40–60 yrs	Intervention: 650 mg of hop tablet/day (contains 100 μg of the active ingredient) (*n* = 60) Control: placebo (*n* = 60)	Iran	GCS and hot flash number	Hop reduced mean GCS score and hot flash numbers (*p* < 0.001). No adverse effects.
Eatemadnia, 2019 [43]	RCT	2 months	80 postmenopausal women aged 45–60 yrs	Intervention: 3 tablets/day of 270–330 μg of *Hypericum perforatum* (*n* = 40) Control: placebo (*n* = 40)	Iran	KI and HDRS	Supplementation reduced the total KI score (*p* < 0.0001) and reduced frequency and intensity of hot flashes (*p* < 0.001). Reduced number experiencing depression (*p* < 0.001).
Erkkola, 2010 [26]	Cross-over RCT study	8 weeks each treatment	36 menopausal women aged 45–60 yrs	Intervention: hop extract (standardised at 100 μg of 8-prenylnaringenin per day) Control: placebo	Finland	KI, VAS, and MRS	Significant reductions for KI (*p* = 0.02) and VAS (*p* = 0.03) and a non-significant reduction (*p* = 0.06) in MRS after 16 weeks. No adverse effects.
Farzaneh, 2013 [20]	RCT	6 weeks	56 menopausal women aged 45–59 yrs	Intervention: 2 capsules/day of evening primrose (500 mg) (*n* = 56) Control: 2 capsules/day of placebo (*n* = 56)	Iran	HFRDIS	The severity but not frequency or duration of hot flashes was reduced (*p* < 0.05) in the intervention group. Of the scales in the HFRDIS, only social activity, relations with others and sexuality improved in the supplement group.
Heyerick, 2006 [25]	Prospective, double-blind RCT	12 weeks	67 menopausal women aged 45–60 yrs	Intervention: 1 capsule/day; group 1 (100 μg of 8-prenylnaringenin; *n* = 20); group 2 (250 μg of 8-prenylnaringenin; *n* = 21) Control: 1 capsule/day of 120 mg of maltodextrin (*n* = 26)	Belgium	KI; diary for hot flushes	Reduced KI after 6 but not 12 weeks in Group 1 (*p* < 0.05). Hot flush score reduced in both intervention groups (*p* < 0.01) after 6 weeks but not 12 weeks.
Hirata, 1997 [44]	RCT	24 weeks	71 postmenopausal women (52.4 ± 6 yrs); FSH levels of >30 mIU/mL	Intervention: 3 capsules/day of dong quai (equivalent to 4.5 g of extract) (*n* = 35) Control: 3 capsules/day of placebo (*n* = 36)	USA	KI; diary for hot flushes	No significant difference in KI or hot flush frequency between two groups.
Howes, 2004 [28]	RCT	6 months	30 postmenopausal women aged >60 yrs	Intervention: 2 tablets/day of aglycone isoflavones from red clover (each containing 25 mg of formononetin, 2.5 mg of biochanin and less than 1 mg of daidzein and genistein) (*n* = 15) Control: placebo (*n* = 15)	Australia	Primary outcome: cognitive function	Intervention group shows improvement in block design, but no other major short-term effects.
Hsu, 2011 [34]	Two-centre, double-blind, RCT	12 months	50 women aged 45–60 yrs, with serum FSH level of 440 mIU/mL	Intervention: 2 sachets/day of Diascorea extracts (containing 12 mg/sachet) (*n* = 25) Control: 2 sachets/day of placebo (*n* = 25)	Taiwan, China	GCS	Intervention group shows reduction in GCS scores after 12 months (*p* < 0.01); significance was specifically observed for psychological parameters like insomnia. No adverse effects.
Jenabi, 2018 [32]	Triple-blinded RCT	2 months	60 postmenopausal women aged 45–55 yrs	Intervention: 2 capsules/day of oral 530 mg valerian capsule (*n* = 30) Control: 2 placebo capsules/day of starch (*n* = 30)	Iran	KI	Severity significantly reduced (*p* = 0.020), and frequency also reduced (*p* = 0.033) in the valerian group. No adverse effects.
Jiang, 2015 [45]	Double-blinded RCT	6 months	48 postmenopausal women aged 45–60 yrs with sleep disturbance	Intervention: 2 tablets/day of black cohosh (about 2.5 mg of extract each) (*n* = 24) Control: 2 tablets/day of placebo (*n* = 24)	China	PSQI; MENQOL	Non-significant polysomnographic changes, with overall medium effect (*p* = 0.051); vasomotor and physical domains of MENQOL improved in intervention group. No adverse effects.
Karimi, 2024 [37]	Triple-blinded RCT	1 month	60 menopausal women aged 40–65 yrs	Intervention: one 500 mg capsule/day of Ocimum basilicum leaf extract (OBLE) (*n* = 38) Control: 1 capsule/day of placebo (*n* = 38)	Iran	MRS	Significant reduction in MRS score (*p* = 0.001) in OBLE group. No adverse effects.
Kazemi, 2021 [22]	RCT	8 weeks	170 post-menopausal women aged 54.7 ± 4.8 yrs	Intervention: two 1000 mg capsules of evening primrose oil/day (*n* = 85) Control: Two 1000 mg capsules of placebo/day (*n* = 85)	Iran	Wiklund Vasomotor Symptom Subscale score	No statistical difference regarding hot flash was observed, but lower frequency and severity of night sweats was seen (*p* < 0.05) in the intervention group.
Lee, 2010 [42]	Double-blinded RCT	12 weeks	87 postmenopausal women aged 40–60 yrs	Intervention: 2 capsules/day of Rexflavone (175 mg) (*n* = 39) Control: 2 capsules/day of placebo (*n* = 41)	Korea	KI	Significant improvement in KI score (*p* < 0.03) in Rexflavone group. No adverse effects.
Lipovac, 2012 [29]	Cross-over RCT study	12 weeks	109 postmenopausal women aged 40 or more	Intervention: 2 capsules/day of 80 mg of red clover isoflavones. Control: 2 capsules/day of placebo	Austria	KI and hot flush diary	Reduced KI score and day/night hot flush frequency after supplementation with active ingredient (*p* < 0.0001). No side effects.
Park, 2016 [40]	Double-blinded RCT	6-week treatment/12-week follow-up	36 women between the ages of 40 and 70 yrs	Intervention: 4 pills/day, each containing 196 mg of BMO-30, a total of 784 mg of natural extract (*n* = 18) Control: 4 pills/day of placebo (*n* = 18)	South Korea	KI	Total KI score significantly lower in the BMO-30 group at 6 (*p* < 0.001) and 12 weeks (*p* = 0.042).
Saberi, 2020 [41]	Parallel, double-blinded RCT	8-week treatment/4-week follow-up	80 women aged 40–60 yrs	Intervention: *Silybum marianum* extract (400 mg/d) (*n* = 40) Control: placebo (*n* = 40)	Iran	GCS; HFRDIS	Significant decrease in GCS and HFRDIS scores in intervention group after 4, 8 and 12 weeks (*p* < 0.001).
Tanmahasamut, 2015 [19]	Double-blinded RCT	12 weeks	54 peri- or postmenopausal Thai women aged at least 40 years	Intervention: 40 mg of black cohosh extract/day (*n* = 27) Control: placebo (*n* = 27)	Thailand	KI; MENQOL	No significant differences between the two groups. No adverse effects.
Yang, 2007 [46]	Double-blinded RCT; intervention group	6 months	200 peri-menopausal women aged 45–55 yrs	Intervention: 200 mg of Pycnogenol daily (*n* = 100) Control: placebo (*n* = 100)	Taiwan, China	WHQ	All climacteric symptoms improved in the Pycnogenol group. No adverse effects.
Zeidabadi, 2020 [31]	Double-blinded RCT	3 months	66 postmenopausal women complaining of menopausal symptoms	Intervention: 3 tablets/day of *Salvia officinalis* tablets (containing 100 mg of *S. officinalis* extract) (*n* = 33) Control: 3 tablets/day of placebo (*n* = 33)	Iran	MRS, PSQI	Total MRS not provided but significant differences were observed in all domains except sexual and urinary factors and exhaustion (*p* < 0.05). Mean score of PSQI significantly decreased by 3.8 units in the intervention group (*p* < 0.05).
Ghavi, 2023 [21]	Triple-blinded RCT	8 weeks	125 participants aged 45–60 yrs	Intervention: fennel group received 30 mg of fennel (*n* = 47); evening primrose oil group received 70 mg–140 mg of gamolenic acid (GLA; *n* = 45) obtained from EPO Control: 2 tablets of placebo/day (*n* = 43)	Iran	MRS and hormonal assay	Mean FSH and oestradiol levels changed significantly in both intervention groups (*p* < 0.001). Fennel but not EPO (*p* < 0.001 and *p* < 0.055, respectively) reduced MRS score after the supplementation.
Hidalgo, 2005 [27]	Double blinded, cross-over RCT	12 weeks	60 postmenopausal women aged >40 years	Intervention: red clover isoflavone supplement (80 mg/day) Control: placebo	Ecuador	KI	KI score decreased after the isoflavone phase (*p* < 0.05) compared with baseline. Two participants withdrew due to adverse effects (headaches).
Mirabi, 2013 [33]	Double-blinded RCT	8 weeks	68 menopausal women with the chief complaint of hot flash aged 45–55 yrs	Intervention: 255 mg of valerian capsules 3 times a day (*n* = 35) Control: placebo with starch (*n* = 33)	Iran	Severity and frequency of hot flashes	Significant reduction in severity (*p* < 0.001) and frequency (*p* < 0.001) in valerian group.
Safdari, 2021 [23]	Triple-blinded RCT	4 weeks	100 menopausal women aged 46–63 yrs	Intervention: two 1 g pearls of EPO daily (*n* = 50) Control: placebo (*n* = 50)	Iran	MRS	A significant reduction in MRS was observed in the intervention group (*p* < 0.001).
Mohammad-Alizadeh-Charandabi, 2013 [17]	Double-blinded RCT	8 weeks	84 early post-menopausal participants aged 45–60 yrs	Intervention: 6.5 mg of dried extract of black cohosh roots daily (*n* = 42) Control: placebo (*n* = 42)	Iran	GCS	Significant reduction in GCS and all subdomains of GCS (*p* < 0.001). No adverse effects.
Bagheriani, 2023 [39]	Double-blinded RCT	8 weeks	90 postmenopausal women aged over 45 years with menopausal hot flashes	Intervention: 500 mg of encapsulated processed rheum ribes twice a day (*n* = 45) Control: starch placebo twice a day (*n* = 45)	Iran	KI	Reduction in mean of hot flashing (*p* < 0.001). No adverse effects reported.
Mahmoudi, 2020 [35]	Double-blinded RCT	21 days	106 postmenopausal women aged 45–65 yrs	Intervention: 250 mg oral jujube seed capsule twice/day (*n* = 53) Control: placebo capsule twice/day (*n* = 53)	Iran	PSQI	Improvement observed in the intervention group (*p*< 0.001), compared with control group (*p* < 0.05) compared with baseline.
Nahidi, 2012 [36]	Double-blinded RCT	8-week treatment/4-week follow-up	90 women aged 45–60 yrs	Intervention: 3 capsules daily containing 330 mg of liquorice extract (*n* = 45) Control: 3 capsules daily containing 330 mg of starch (*n* = 45)	Iran	Hot flashes	Frequency and severity of hot flashes decreased in the intervention group (*p* < 0.05); effects lasted for 2 weeks.
Wilfried, 2021 [30]	Double-blinded RCT	4 weeks	80 menopausal women from 48 to 65 yrs	Intervention: Menosan^®^ tablets containing 3400 mg of ethanolic extract of freshly harvested *Salvia officinalis* L. (*n* = 40) Control: placebo (*n* = 40)	Germany	MRS, HFS, qEEG, and sleep quality	Salvia off. reduced MRS by 39.2% (*p* = 0.002); HFS score decreased by 55.3% (*p* = 0.028). Further positive impact seen on sleep quality, discontent and fatigue (*p* < 0.05). No adverse effects reported.
Schellenberg, 2012 [18]	Double-blinded RCT	12 weeks	180 women >40 yrs (ITT *n* = 166)	Intervention: (b) 6.5 mg of Ze 450, (low dose; LD; *n* = 57), and (c) 13 mg of Ze 450 (high dose; HD; *n* = 55). Control: (a) Placebo (*n* = 54)	Germany	KI, QoL, and safety	Patients receiving Ze 450 showed a significant reduction in total KI (*p* = 0.0003 and *p* = 0.0057) and QoL (*p* < 0.0001 and 0.06) with low and high doses; QoL. No safety concerns.

Abbreviations: RCT: randomised controlled trial; KI: Kupperman’s Index; MENQOL: Menopause-Specific Quality of Life Questionnaire; GCS: Greene Climacteric Scale; HDRS: Hamilton Depression Rating Scale; MRS: Menopause Rating Scale; VAS: Visual Analogue Scale; HFRDIS: Hot Flash-Related Daily Interference Scale; FSH: follicle-stimulating hormone; PSQI: Pittsburgh Sleep Quality Index; WHQ: Women’s Health Questionnaire; EPO: evening primrose oil; HFS: Hot Flush Severity score; and qEEG: quantitative electroencephalography risk of bias.

## Data Availability

No new data were created or analyzed in this study. Data sharing is not applicable to this article.

## Supplementary Materials

The following supporting information can be downloaded at: https://www.mdpi.com/article/10.3390/nu18132037/s1.
