# Supplementary material for: The Effectiveness of Non-Soy Oral Herbal Supplements for Menopausal Symptoms: A Systematic Review
Source: Nutrients. 2026 Jun 23;18(13):2037. doi: 10.3390/nu18132037 (PMC13363246; doi:10.3390/nu18132037)
Supplement: Supplementary file 1 [file nutrients-18-02037-s001.zip › nutrients-4310191-supplementary.pdf]

Supplemental information 1:

Search String Pubmed:

("Herbal Medicine"[MeSH Terms] OR "Phytotherapy"[MeSH Terms] OR "herbal*"[Title/Abstract] OR "botan*"[Title/Abstract] OR "phytotherap*"[Title/Abstract] OR "supplement*"[Title/Abstract] OR "Evening Primrose Oil"[Title/Abstract] OR "Oenothera biennis"[Title/Abstract] OR "gamma linolenic acid"[Title/Abstract] OR "GLA"[Title/Abstract] OR "Black cohosh"[Title/Abstract] OR "Actaea racemosa"[Title/Abstract] OR "Cimicifuga racemosa"[Title/Abstract] OR "Red clover"[Title/Abstract] OR "Trifolium pratense"[Title/Abstract] OR "isoflavone*"[Title/Abstract] OR "genistein"[Title/Abstract] OR "daidzein"[Title/Abstract] OR "soy"[Title/Abstract] OR "Glycine max"[Title/Abstract] OR "hops"[Title/Abstract] OR "Humulus lupulus"[Title/Abstract] OR "8-prenylnaringenin"[Title/Abstract] OR "8-PN"[Title/Abstract] OR "sage"[Title/Abstract] OR "Salvia officinalis"[Title/Abstract] OR "dong quai"[Title/Abstract] OR "Angelica sinensis"[Title/Abstract] OR "chasteberry"[Title/Abstract] OR "Vitex agnus-castus"[Title/Abstract] OR "ginseng"[Title/Abstract] OR "Panax"[Title/Abstract] OR "maca"[Title/Abstract] OR "Lepidium meyenii"[Title/Abstract] OR "flaxseed"[Title/Abstract] OR "linseed"[Title/Abstract] OR "lignan*"[Title/Abstract] OR "secoisolariciresinol diglucoside"[Title/Abstract] OR "wild yam"[Title/Abstract] OR "Dioscorea"[Title/Abstract] OR "Pueraria mirifica"[Title/Abstract] OR "kudzu"[Title/Abstract] OR "pycnogenol"[Title/Abstract] OR "French maritime pine"[Title/Abstract] OR "resveratrol"[Title/Abstract]) AND ("Menopause"[MeSH Terms] OR "Postmenopause"[All Fields] OR "Perimenopause"[All Fields] OR "Climacteric"[All Fields]) AND ("Sleep"[MeSH Terms] OR "Insomnia"[All Fields] OR "Sleep Quality"[All Fields] OR ("Neurological Symptoms"[All Fields] OR "Mood"[All Fields] OR "Cognition"[All Fields]) OR ("Vasomotor Symptoms"[All Fields] OR "Hot Flashes"[All Fields] OR "Night Sweats"[All Fields]))

Search String Embase:

('exp herbal medicine/' OR 'exp phytotherapy/' OR herbal*:ti,ab,kw OR botan*:ti,ab,kw OR phytotherap*:ti,ab,kw OR supplement*:ti,ab,kw OR 'Evening primrose oil':ti,ab,kw OR 'Oenothera biennis':ti,ab,kw OR 'gamma linolenic acid':ti,ab,kw OR GLA:ti,ab,kw OR 'black cohosh':ti,ab,kw OR 'Actaea racemosa':ti,ab,kw OR 'Cimicifuga racemosa':ti,ab,kw OR 'red clover':ti,ab,kw OR 'Trifolium pratense':ti,ab,kw OR isoflavone*:ti,ab,kw OR genistein:ti,ab,kw OR daidzein:ti,ab,kw OR soy:ti,ab,kw OR 'Glycine max':ti,ab,kw OR hops:ti,ab,kw OR 'Humulus lupulus':ti,ab,kw OR '8-prenylnaringenin':ti,ab,kw OR '8-PN':ti,ab,kw OR sage:ti,ab,kw OR 'Salvia officinalis':ti,ab,kw OR 'dong quai':ti,ab,kw OR 'Angelica sinensis':ti,ab,kw OR chasteberry:ti,ab,kw OR 'Vitex agnus-castus':ti,ab,kw OR ginseng:ti,ab,kw OR Panax:ti,ab,kw OR maca:ti,ab,kw OR 'Lepidium meyenii':ti,ab,kw OR flaxseed:ti,ab,kw OR linseed:ti,ab,kw OR lignan*:ti,ab,kw OR 'secoisolariciresinol diglucoside':ti,ab,kw OR 'wild yam':ti,ab,kw OR Dioscorea:ti,ab,kw OR 'Pueraria mirifica':ti,ab,kw OR kudzu:ti,ab,kw OR pycnogenol:ti,ab,kw OR 'French maritime pine':ti,ab,kw OR resveratrol:ti,ab,kw) AND('exp menopause/' OR postmenopause:ti,ab,kw OR perimenopause:ti,ab,kw OR climacteric:ti,ab,kw) AND ('exp sleep/' OR'exp insomnia/' OR 'exp sleep disorder/' OR 'exp cognition/' OR 'exp mood disorder/' OR 'exp hot flush/' OR 'exp vasomotor symptom/' OR sleep quality:ti,ab,kw OR insomnia:ti,ab,kw OR (neurological symptoms:ti,ab,kw OR mood:ti,ab,kw OR cognition:ti,ab,kw) OR (vasomotor symptoms:ti,ab,kw OR hot flash*:ti,ab,kw OR night sweat*:ti,ab,kw)) NOT ([animals]/lim NOT [humans]/lim)

Search String PsycINFO:

((exp Herbal Medicine/ OR exp Phytotherapy/ OR herbal*.ti,ab,kw. OR botan*.ti,ab,kw. OR phytotherap*.ti,ab,kw. OR supplement*.ti,ab,kw. OR "Evening primrose oil".ti,ab,kw. OR "Oenothera biennis".ti,ab,kw. OR "gamma linolenic acid".ti,ab,kw. OR GLA.ti,ab,kw. OR "black cohosh".ti,ab,kw. OR "Actaea racemosa".ti,ab,kw. OR "Cimicifuga racemosa".ti,ab,kw. OR "red clover".ti,ab,kw. OR "Trifolium pratense".ti,ab,kw. OR isoflavone*.ti,ab,kw. OR genistein.ti,ab,kw. OR daidzein.ti,ab,kw. OR soy.ti,ab,kw. OR "Glycine max".ti,ab,kw. OR hops.ti,ab,kw. OR "Humulus lupulus".ti,ab,kw. OR "8-prenylnaringenin".ti,ab,kw. OR "8-PN".ti,ab,kw. OR sage.ti,ab,kw. OR "Salvia officinalis".ti,ab,kw. OR "dong quai".ti,ab,kw. OR "Angelica sinensis".ti,ab,kw. OR chasteberry.ti,ab,kw. OR "Vitex agnus-castus".ti,ab,kw. OR ginseng.ti,ab,kw. OR Panax.ti,ab,kw. OR maca.ti,ab,kw. OR "Lepidium meyenii".ti,ab,kw. OR flaxseed.ti,ab,kw. OR linseed.ti,ab,kw. OR lignan*.ti,ab,kw. OR "secoisolariciresinol diglucoside".ti,ab,kw. OR "wild yam".ti,ab,kw. OR Dioscorea.ti,ab,kw. OR "Pueraria mirifica".ti,ab,kw. OR kudzu.ti,ab,kw. OR pycnogenol.ti,ab,kw. OR "French maritime pine".ti,ab,kw. OR resveratrol.ti,ab,kw.) AND (exp Menopause/ OR postmenopause.ti,ab,kw. OR perimenopause.ti,ab,kw. OR climacteric.ti,ab,kw.) AND (exp Sleep/ OR exp Insomnia/ OR exp Cognition/ OR exp Mood/ OR sleep quality.ti,ab,kw. OR insomnia.ti,ab,kw. OR neurological symptoms.ti,ab,kw. OR mood.ti,ab,kw. OR cognition.ti,ab,kw. OR vasomotor symptoms.ti,ab,kw. OR hot flash*.ti,ab,kw. OR night sweat*.ti,ab,kw.))

Search String Scopus:

(TITLE-ABS-KEY(herbal* OR botan* OR phytotherap* OR supplement* OR "Evening primrose oil" OR "Oenothera biennis" OR "gamma linolenic acid" OR GLA OR "black cohosh" OR "Actaea racemosa" OR "Cimicifuga racemosa" OR "red clover" OR "Trifolium pratense" OR isoflavone* OR genistein OR daidzein OR soy OR "Glycine max" OR hops OR "Humulus lupulus" OR "8-prenylnaringenin" OR "8-PN" OR sage OR "Salvia officinalis" OR "dong quai" OR "Angelica sinensis" OR chasteberry OR "Vitex agnus-castus" OR ginseng OR Panax OR maca OR "Lepidium meyenii" OR flaxseed OR linseed OR lignan* OR "secoisolariciresinol diglucoside" OR "wild yam" OR Dioscorea OR "Pueraria mirifica" OR kudzu OR pycnogenol OR "French maritime pine" OR resveratrol)) AND (TITLE-ABS-KEY(menopause OR postmenopause OR perimenopause OR climacteric)) AND (TITLE-ABS-KEY(sleep OR insomnia OR "sleep quality" OR "neurological symptoms" OR mood OR cognition OR "vasomotor symptoms" OR "hot flash*" OR "night sweat*"))
